# ADHD and perceptual learning: insights from the intermixed-blocked effect

**DOI:** 10.3389/fpsyg.2026.1645399

**Published:** 2026-05-04

**Authors:** Naiara Arriola, Eugenia Marin-Garcia, Yeray Mera, Gabriel Rodríguez

**Affiliations:** Facultad de Psicología, University of the Basque Country (EHU), Donostia/San Sebastián, Spain

**Keywords:** ADHD, awareness, implicit learning, interleaved-blocked, intermixed-blocked, perceptual learning

## Abstract

Perceptual learning, which is typically considered an implicit and automatic process, has received little attention in the study of attention deficit hyperactivity disorder (ADHD). In this study, we investigated whether children and adolescents with ADHD show intact perceptual learning using the intermixed-blocks effect, whereby alternating exposure to similar stimuli improves subsequent discrimination compared to block exposure. Eighty-eight participants aged 8 to 16 years (44 with ADHD and 44 controls) completed a flower counting task while incidentally exposed to two types of visually similar flowers that differed only in the number of petals, without being informed of this distinction. In the interleaved condition, the two types of flowers were alternated within each screen; in the blocked condition, only flowers of one type were presented during the first half of the task and all flowers of the other type during the second half. Discrimination was assessed using a no-prior-warning same-different test. The results showed a robust intermixed-blocked effect in both groups, indicating that implicit learning is preserved in ADHD. However, while control participants in the intermixed group often identified the specific distinguishing feature, participants in the same condition but with ADHD more frequently failed to do so, despite correctly judging the stimuli as different. This dissociation suggests that implicit perceptual learning is preserved in ADHD, but that explicit access to learned information may be impaired. These findings highlight the value of perceptual learning paradigms for unravelling automatic and controlled processes in neurodevelopmental conditions.

## 1 Introduction

Attention Deficit Hyperactivity Disorder (ADHD) is one of the most commonly diagnosed neurodevelopmental disorders in children, with global prevalence rate around 5% (e.g., Polanczyk et al., 2007). The primary neurocognitive deficits of ADHD are considered to be impulsivity, hyperactivity, and inattention (American Psychiatric Association, 2013). A widely accepted explanation for this disorder is that it is caused by a deficit in the executive function domain (EF; also called executive control or cognitive control) (e.g., Barkley, 1997; Biederman et al., 2004; Castellanos et al., 2006). EF is usually characterized as a complex system comprising three interactive sub-functions (e.g., Lehto et al., 2003; Miyake et al., 2000): *inhibition* (including *behavioural* and *interference control* via *selective attention*), *working memory*, and *cognitive flexibility* (also called *set shifting*). These executive functions are essential for higher-order cognitive processes such as reasoning, problem solving, and planning (e.g., Collins and Koechlin, 2012). They are therefore also crucial for mental and physical health, since they support academic and professional success, as well as emotional and adaptative functioning in social situations [see reviews by Barkley (2001) and Hervey et al. (2004)].

An implication of the hypothesis that ADHD is caused by EF deficits is that *implicit learning* processes should remain intact in the ADHD population. Implicit learning is characterized by a set of automatic, associative, non-conscious, and unintentional processes, in contrast to the conscious, deliberate, and reflective learning processes that are thought rely on EF (e.g., Barrett et al., 2004; Reber, 1967). This implication has been tested in previous studies using a variety of procedures, which have yielded inconsistent results. Two studies using artificial grammar learning tasks reported opposite findings. Rosas et al. (2010) found that children with ADHD (aged 6–11 years) performed better than non-ADHD controls, whereas Domuta and Péntek (2000); with children (aged 5–7 years) reported the opposite pattern.

A second group of studies employed the serial reaction time task, and also produced inconclusive results. Pedersen and Ohrmann (2018) found unimpaired performance in adult ADHD participants, but this pattern was only partially replicated in three further studies involving children and adolescents. One study showed intact performance in ADHD participants aged 10–18, (Vloet et al., 2010). However, two other studies (Barnes et al., 2010; Karatekin et al., 2009; with participants aged 7–12, and 8–19, respectively) reported compromised performance in the ADHD groups, although only in some specific aspects of the task (e.g., fewer oculomotor anticipations and more inconsistent learning trajectories). To explain these mixed results, Pedersen and Ohrmann (2018) proposed that implicit learning *per se* is likely unimpaired in ADHD at any age, but EF deficits—particularly in response inhibition and/or error monitoring—may interfere with the expression of that learning, especially in children (e.g., Barnes et al., 2010; Karatekin et al., 2009).

Taking these results together, the status of implicit learning processes in ADHD remains uncertain. Importantly, previous studies have mostly focused on procedural and conceptual implicit learning (e.g., through serial reaction and artificial grammar tasks, respectively). To the best of our knowledge, a different sort of implicit learning—*perceptual learning*—has not yet been explored in this population. The aim of the present study was addressing this gap by investigating perceptual learning in children and adolescents with ADHD.

From a wide perspective, perceptual learning is considered as a set of processes triggered by mere stimulus exposure that change the way the stimuli are perceived. One well-known instance of perceptual learning is that certain exposure schedules can facilitate later discrimination of similar stimulus [see reviews in Hall (1991) and Mitchell and Hall (2014)]. Within this domain, we will focus on the so-called *intermixed-blocked* effect (I/B effect; also referred to as the *interleaved-blocked* effect) which has been extensively documented (e.g., Angulo and Alonso, 2012, 2014; Dwyer et al., 2004; Mundy et al., 2007; Recio et al., 2016; Rodríguez and Angulo, 2014; Rodríguez and Rodríguez-San Juan, 2025). This effect refers to the finding that exposure schedules including frequent alternations of similar stimuli (e.g., AX, BX, BX, AX, AX, BX…), lead to better subsequent discrimination than blocked presentations (e.g., AX, AX, AX… BX, BX, BX…). Although some demonstrations of the I/B effect in the human visual domain have been attributed to top-down attentional mechanisms (e.g., Lavis and Mitchell, 2006; Mackintosh, 2009; Recio et al., 2016; Hall, 1991; Rodríguez and Rodríguez-San Juan, 2025), more recent studies have introduced procedures that reveal a clear role for automatic, non-conscious and unintentional processes (Rodríguez et al., 2021; Rodríguez and Rodríguez-San Juan, 2025).

**Figure 1:**
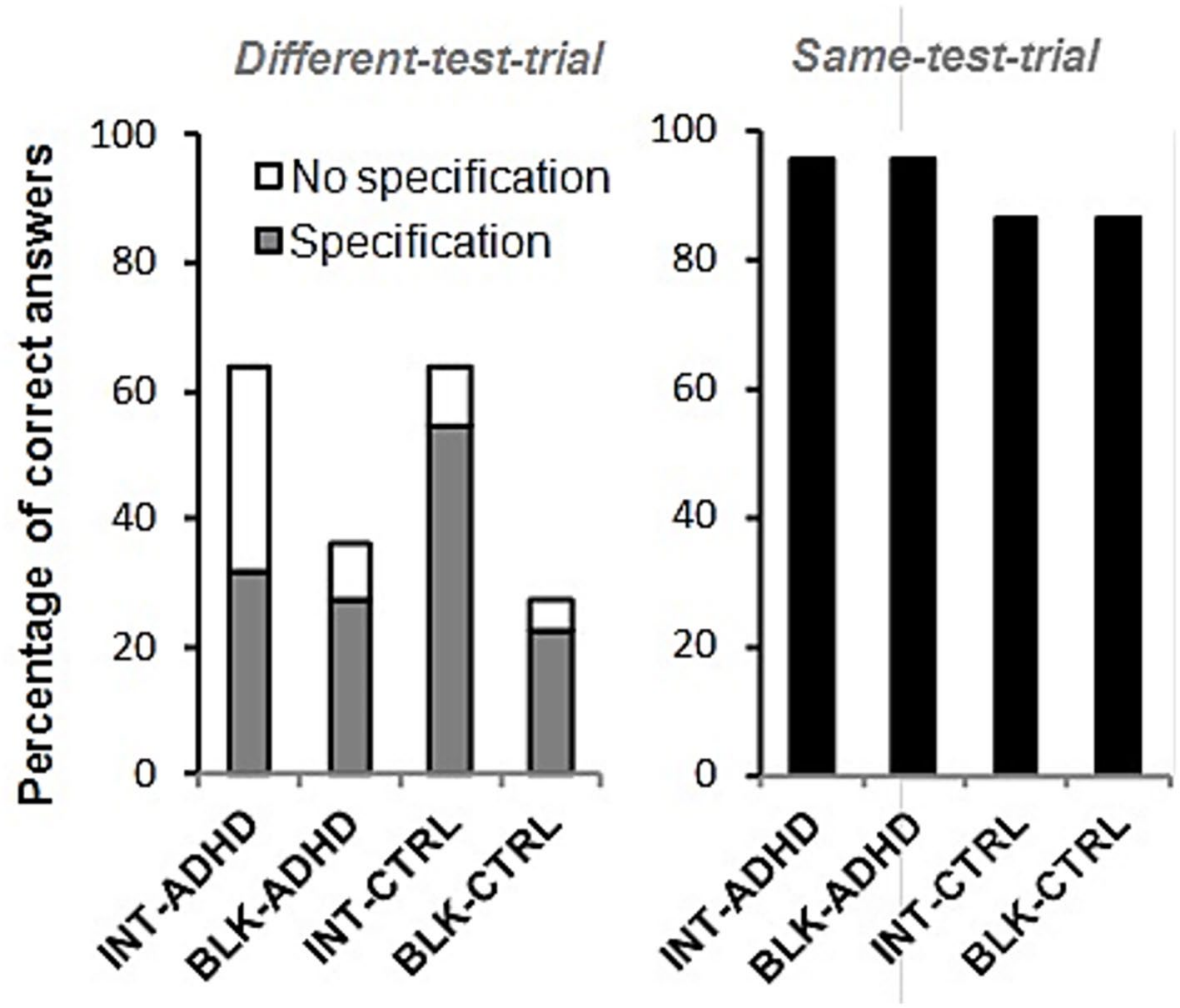
Percentage of correct responses in the different-test-trial (left panel) and in the same-test-trial (right panel) for each experimental group. In the different-test-trial (left panel), the total height of each bar reflects the percentage of participants who correctly judged the stimuli as “different.” This total is divided into two components: the gray area represents responses in which participants explicitly identified the distinguishing feature (“Specification”), and the white area represents responses that judged the stimuli as different without specifying the difference (“No Specification”). The right panel shows the percentage of correct “same” responses in the same-test-trial.

In the present study, we exploited one of those procedures (Rodríguez and Rodríguez-San Juan, 2025) to examine whether an I/B effect mediated by such automatic and non-conscious mechanisms could also be found in children/adolescents with ADHD. The experiment included two groups of participants aged 8–16; one diagnosed with ADHD, and one matched control group. Participants were instructed that they would be exposed to a series of visual displays, each containing several flower images, and that their task was to mentally count, as accurate as possible, the total number of flowers presented across all displays. Crucially, they were not informed that two types of flowers would be presented, differing only in a specific feature (the number of petals) (see Figure 1).

Within each ADHD condition (ADHD vs. Control), participants were assigned to one of two preexposure conditions. In the intermixed condition (ADHD-INT and Ctrl-INT), AX and BX exemplars appeared mixed within each display, leading participants to unintentionally alternate between the two types while performing the counting task. In the blocked condition (ADHD-BLK, Ctrl-BLK) participants were exposed to all exemplars of one stimulus (e.g., AX) in the first half of displays, followed by all exemplars of the other (e.g., BX) in the second half. Thus, in this blocked condition only one transition occurred between stimulus types.

After preexposure, participants were asked to report their final count and then completed two test trials (the Different-test-trial, and the Same-test-trial) assessing their ability to differentiate the stimuli. At the beginning of this test phase, they were merely informed that, on each of these trials, two images would be shown in quick succession. After preexposure, participants completed two test trials assessing their ability to differentiate the stimuli.

**Figure 2:**
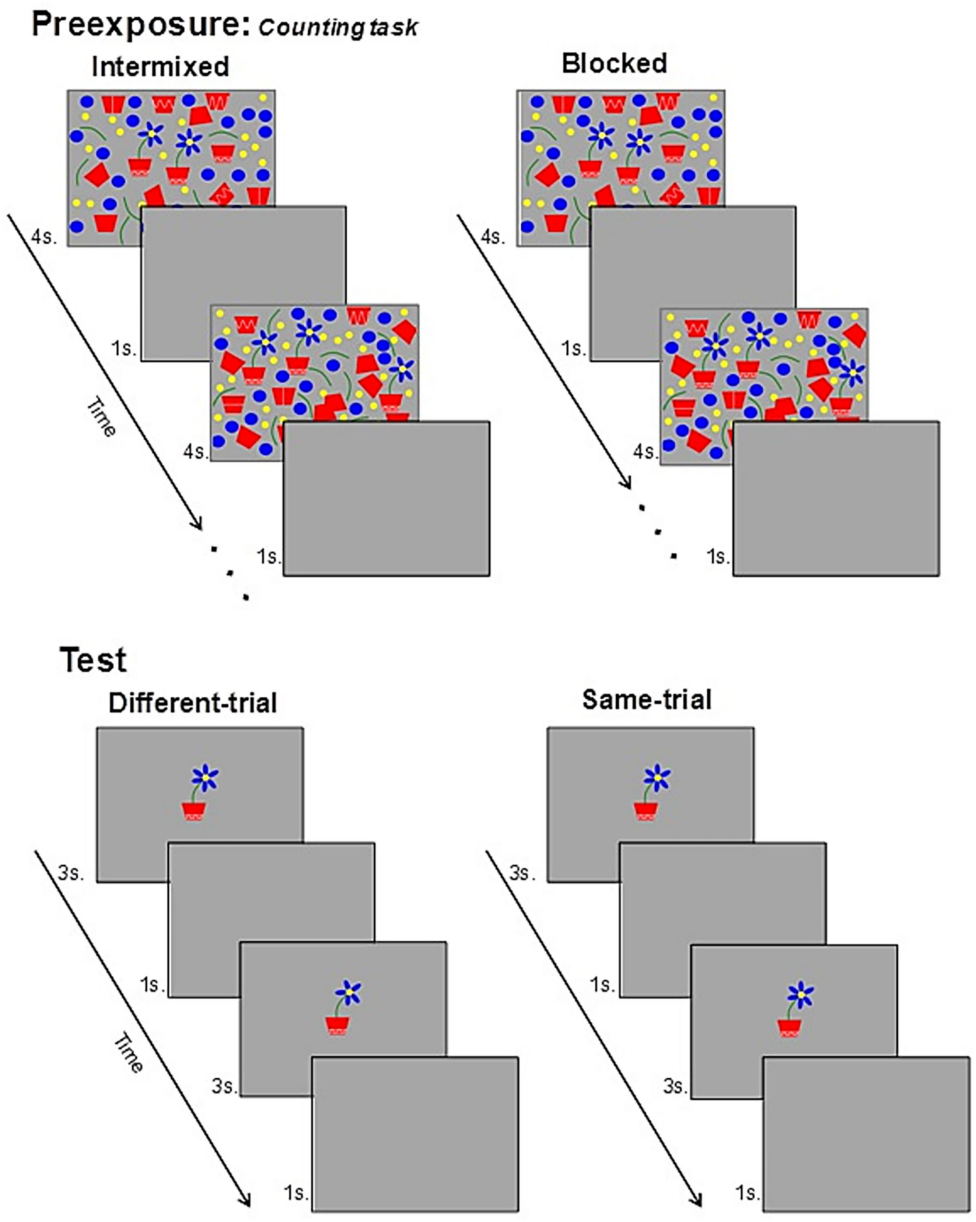
Example of the presentations of the target stimuli AX and BX (flowers with 5 or 6 petals, counterbalanced), during the pre-exposure and test phases. INT groups (INT-ADHD, INT-Ctrl) received intermixed presentations to the target stimuli, following a strict alternating sequence of AX and BX (or vice versa), from left to right in each pre-exposure display. BLK groups (BLK-ADHD, BLK-Ctrl), however, received exposure to the stimuli in blocks, with all the exemplars of one of the targets (e.g., AX) presented in the first pre-exposure displays and all the exemplars of the other target (e.g., BX) being presented in the remaining displays. On each of the test trials, participants in both groups were exposed to two stimuli, one after the other (separated by a blank display). In the different trial the two stimuli presented were different and in the same trial the stimuli were the same.

On the Different-test-trial, an exemplar of one of the two types (e.g., AX) was briefly presented followed by an exemplar of the other type (e.g., BX) (with a blank screen in between to avoid a pop-up effect; see Figure 2). Participants were then asked to judge whether the images were the same or different and, if different, to specify the nature of the difference. A second Same-test-trial followed, identical in structure, but presenting two identical exemplars (e.g., AX-AX).

Several features of this procedure are worth highlighting (for a full discussion, see Rodríguez and Rodríguez-San Juan, 2025). First, the instructions omitted any exhorting for searching differences between the stimuli during the preexposure, but still provided a clear goal: counting targets. These instructions likely engaged top-down attention to features common to the targets, not their differences. Therefore, any post-test improvements in discrimination should reflect incidentally triggered (i.e., automatic and involuntary) learning mechanisms. Second, this procedure allows us to differentiate between implicit and explicit forms of the I/B effect. If improved discrimination after intermixed exposure is accompanied by the ability to identify the distinguishing feature, the effect may be considered *explicit*. If no such identification occurs, the effect is interpreted as *implicit*.

Based on our prior findings (Rodríguez and Rodríguez-San Juan, 2025), we formulated separate predictions for overall discrimination performance and explicit feature identification. Overall discrimination performance was assessed by the proportion of correct “different” judgments in the Different-test-trial, regardless of whether participants could specify the distinguishing feature. If automatic, associative mechanisms are preserved in ADHD, we expected comparable intermixed-blocked effects in both groups—that is, higher discrimination accuracy in intermixed vs. blocked conditions for both ADHD and control participants.

However, we predicted group differences in explicit feature identification. When participants correctly judged stimuli as “different,” we also assessed whether they could specify the distinguishing feature (number of petals). This ability is assumed to rely on higher-order cognitive functions such as working memory and metacognitive awareness, which are known to be compromised in ADHD. Therefore, we predicted that intermixed exposure would enhance explicit identification in controls (Ctrl-INT > Ctrl-BLK) but not in the ADHD group (ADHD-INT ≈ ADHD-BLK).

Finally, in the Same-test-trial, we expected uniformly high accuracy across all groups, consistent with previous studies (e.g., Lavis and Mitchell, 2006; Rodríguez and Rodríguez-San Juan, 2025). This would support the interpretation that the responses of the participants in the Different-test-trial were based on genuine perceptual discrimination, rather than response bias or random guessing.

## 2 Methods

### 2.1 Participants

A total of 88 participants took part in the study: 44 clinically diagnosed with ADHD and 44 matched controls. Participants were matched on gender, age, and educational level. Each group consisted of 22 children and adolescents (aged 8 to 16 years), with the following age distribution: three participants aged 8, three aged 9, three aged 10, two aged 11, three aged 12, two aged 13, four aged 14, one aged 15, and one aged 16. Each group comprised 12 boys and 10 girls. All participants had normal or corrected-to-normal vision. They agreed to participate voluntarily, without any form of compensation, after their legal guardians provided informed consent. The sample was recruited through convenience sampling, as access to children and adolescents with a formal ADHD diagnosis within this age range was limited. Although this may have reduced statistical power, it did not compromise the main rationale or theoretical interpretability of the experimental comparisons, which aimed to assess a possible dissociation between the I/B effect (i.e., the mere ability to differentiate stimuli) and the ability to make explicit the distinguishing feature.

The *Research Ethics Committee of the University of the Basque Country* (CEISH) approved the protocol of the study.

Participants in the ADHD condition were recruited through local ADHD associations affiliated with the Spanish Federation of ADHD Support Associations (FEAADAH; *Federación Española de Asociaciones de Ayuda al Déficit de Atención e Hiperactividad*) and had received a formal diagnosis from qualified mental health professionals. Some of these participants were under stimulant medication, either methylphenidate or mixed amphetamine salts (14 in the ADHD-INT group, and 17 in the ADHD-BLK group). The proportion of medicated participants did not differ significantly between the two ADHD groups, nor did the average time elapsed between the last dose and the beginning of the experiment. Control participants were recruited through structured outreach to families within the university community, including staff networks and personal referrals, ensuring age and educational level matching with the ADHD group.

### 2.2 Apparatus and stimuli

Participants were tested individually on a 17-inch computer monitor, seated approximately 50 cm away from the screen. Stimuli were presented against a uniform gray background. The two target stimuli are shown in Figure 1: two images of a flower planted in a flowerpot, differing only in the number of petals (five or six). One of these two distinctive features was assigned to A and the other to B for half of the participants, while for the other half, this assignment was reversed.

### 2.3 Procedure

Within each diagnostic condition, participants were assigned to preexposure conditions in a manner that ensured balanced distribution of age and gender across groups. Prior to assignment, participant lists including age and gender information had been compiled, allowing the experimenters to organize balanced groups. When multiple participants of the same age and gender were available, random assignment was applied within that stratum. This resulted in four groups: ADHD-INT (*n* = 22), Ctrl-INT (*n* = 22), ADHD-BLK (*n* = 22), and Ctrl-BLK (*n* = 22). The procedure consisted of two main phases: a *preexposure phase* and a *test phase*.

In the preexposure phase, participants received the following instructions (in Spanish): *Next, you will be exposed to an automatic sequence of screens. On these screens, there will be several flowers planted in flowerpots. Your task is to mentally count, as accurately as possible, how many flowers are presented, in overall, across the entire succession of screens. Press the START button when you will be ready to begin the task*.

Following the procedure of Rodríguez and Rodríguez-San Juan (2025), in which these parameters yielded a robust I/B effect with the same stimuli, this phase involved the presentation of 14 screens, each showing a variable number of target stimuli (flowers) accompanied by visual distractors, for 4 s (see Figure 1 for examples). A gray background was used throughout. Each screen was followed by a 1-s blank interval. To avoid making the counting task too simple, the number of targets per screen varied according to the sequence: 2, 3, 4, 2, 3, 2, 2, (repeated twice, yielding a total of 36 targets). Although the positions of the targets and the distractors varied from screen to screen, they were fixed across participants for each screen.

Groups differed in the distribution pattern of AX and BX stimuli across the screens. For participants in the INT groups, the targets were presented in a strict alternating order between AX and BX (e.g., AX, BX, and AX), and this alternation was maintained across screens. To support this structure and facilitate natural left-to-right visual scanning—similar to reading—when the last target on the right side of a screen was AX, the first target on the left side of the next screen was BX. Participants in the BLK group received on the first screens all the presentations of one of the targets (AX or BX) and then, on the remaining screens, all the presentations of the other target. The order of presentation (AX-first or BX-first) was counterbalanced across participants within each group. Importantly, participants were not informed of the existence of two types of flowers or instructed to attend to differences between them, which ensured that any facilitation in subsequent discrimination could be attributed to incidental learning. The instructions emphasized accurate counting, thereby engaging goal-directed attention to the targets without directing attention to their distinguishing features.

When the preexposure phase ended, the *test phase* began. All the participants received the following instructions: *Please, report now the result of your counting.* Immediately after typing their answers into the computer, all participants were presented on a screen with the following instructions for the test phase*: Next, two images will be quickly presented, one after the other. You just have to look at them. Press the START button when you will be ready*.

When the participants pressed the button the first test trial began. This was the Different-test-trial and had the following structure: blank screen- > AX- > blank screen- > BX- > blank screen. Each stimulus was presented for 3 s, and they were intercalated between blank screens of 1 s. Immediately after the presentation of the last blank screen, all the participants were tested in their ability to differentiate the stimuli. Specifically, participants were presented with a screen with the following instructions and questions: *The two images that you have just seen … were they the same or different? In the case that your answer will be “different,” please specify accurately in what aspect, or aspects, you believe that they differed. If you think that the images were identical simply answer “same*.” Participants typed their answers in an empty textbox under these instructions/questions.

After completing their response, participants proceeded to the second test trial, the Same-test-trial, which was identical in structure but presented the same target stimulus (AX) twice following this structure: blank screen- > AX- > blank screen- > AX- > blank screen. At the end of this trial, participants received the same instructions and questions that they received on the previous different-test trial, and typed their answers.

### 2.4 Data treatment and analysis

Two independent raters, blinded to both the hypotheses and group assignments, evaluated the responses provided by the participants in the counting and differentiation tasks. Prior to coding, the raters were provided with the total number of stimuli presented and shown examples of the AX and BX stimuli, with the distinguishing feature (number of petals) explicitly indicated.

Counting accuracy was evaluated according to two scoring criteria. The strict criterion considered as correct only those responses that exactly matched the number of target stimuli presented (36). A more lenient criterion considered as correct any response within a 10% deviation range (32–40), to reflect meaningful engagement with the task even in the absence of perfect precision.

Responses on the Different-test-trial were classified into three exclusive categories: (1) “same”—judging the stimuli as identical, (2) “different–no specification”—judging them as different without identifying the critical feature, and (3) “different–specification”—correctly identifying the number of petals as the distinguishing feature. Responses on the Same-test-trial, were coded as correct when identifying the stimuli as identical, and incorrect otherwise.

Interrater reliability was perfect for all coded variables (Cohen’s *κ* = 1.00, *p* < 0.001). Categorical data were analyzed using chi-square (*χ*^2^) tests. When significant results were obtained, effect sizes were calculated using Cramér’s *V*, and 95% confidence intervals were reported. Adjusted standardized residuals were examined for *post hoc* comparisons to determine which cells deviated most from expected frequencies under the null hypothesis. Residuals with an absolute value greater than ±1.96 were interpreted as statistically significant (*α* = 0.05, two-tailed).

### 2.5 Transparency and openness

The datasets analyzed for this study can be found in the Open Science Framework (OSF) repository: https://osf.io/76fqn/. All materials are available from the corresponding author upon request. Data were analyzed using SPSS v. 26. The design and analysis of this study were not preregistered.

## 3 Results

### 3.1 Counting task

The performance of the participants in the counting task was relatively low but similar in all the groups. The percentages of participants who reported the exact result (36 targets) were: 31.8, 36.4, 45.5, and 36.4% in the groups ADHD-INT, ADHD-BLK, Ctrl-INT and Ctrl-BLK, respectively. The percentages of participants who reported the exact or an approximate result (between 30 and 40) in the counting were: 45.5, 59.1, 63.6, and 54.5% in the same groups, respectively. Chi-square analyses revealed no significant differences in the distribution of correct answers across the groups, both for exact responses, *χ*^2^(3) = 0.92, *p* = 0.82, and for approximate responses, *χ*^2^(3) = 1.61, *p* = 0.66. It is worth noting that the ease with which the younger participants in the present sample followed the counting task was lower than that observed in our previous study (Rodríguez and Rodríguez-San Juan, 2025) with older participants (mean age approximately 18 years), in which more than 90% of participants reported an approximate result within the correct range.

As some participants in the ADHD condition were under medication and others were not, we explored the possibility that medication status could have influenced performance in the counting task. No significant correlations were found between medication status (on vs. off) and either of the two performance measures in the counting task, *r*(44) = 0.045, *p* = 0.77 for exact responses, and *r*(44) = 0.079, *p* = 0.61 for approximate responses. Furthermore, among participants who were under medication in this condition, no significant correlation was observed between the time interval from medication intake to the moment of testing and performance on the counting task, *r*(31) = −0.069, *p* = 0.71 for exact responses, and *r*(31) = −0.030, *p* = 0.87 for approximate responses.

### 3.2 Perceptual learning task

#### 3.2.1 Different-test-trial

Figure 2 illustrates the percentage of correct responses in the Different-test-trial for each experimental group. The left panel of the figure shows the percentage of participants who judged the stimuli as different, regardless of whether they explicitly identified the specific difference (Specification) or not (No Specification). As can be clearly observed, an intermixed-blocked (I/B) effect emerged in both the ADHD and control (non-ADHD) groups, with higher percentages of correct responses in the intermixed (INT) groups compared to the blocked (BLK) groups in both conditions. This general I/B effect was confirmed by a hierarchical log-linear analysis exploring the effects of *ADHD condition* (ADHD vs. Control), *exposure schedule* (INT vs. BLK), and *response accuracy* (Correct vs. Incorrect). An initial comparison of the three-way table with the model of complete independence confirmed that the variables were not mutually independent, G^2^(4) = 9.51, *p* < 0.05. Building on this, the hierarchical log-linear analysis showed a robust Exposure × Accuracy interaction, G^2^(1) = 9.09, *p* = 0.003, Cramér’s *V* = 0.32, indicating that intermixed exposure (INT) reliably increased the proportion of correct detections relative to blocked exposure (BLK). Neither the ADHD × Exposure, G^2^(1) = 0.24, *p* = 0.62, nor the ADHD × Accuracy, G^2^(1) = 0.42, *p* = 0.52, interaction reached significance, and the three-way interaction (ADHD × Exposure × Accuracy) was negligible, G^2^(1) = 0.22, *p* = 0.64.

An inspection of the pattern of responses shown in Figure 2 suggests the existence of possible qualitative differences in the I/B effect in the ADHD and control conditions. More specifically, within the intermixed (INT) condition, children with ADHD produced a markedly different response profile from controls. Of the 14 correct detections in the INT-ADHD group, only half (7/14) included an explicit identification of the distinguishing feature, whereas 12 of 14 controls did so. A 2 × 2 Chi-square test (INT-ADHD vs. INT-Control × Specification vs. No-Specification) yielded *χ*^2^(1) = 4.09, *p* = 0.043; but with Yates’s continuity correction the effect became a trend, *χ*^2^(1) = 2.62, *p* = 0.106, Cramér’s *V* = 0.31. Adjusted standardized residuals indicated an over-representation of No-Specification responses in the INT-ADHD group (*z* = +2.02) and an under-representation in the INT-Control group (*z* = −2.02). These data suggest that, although both groups benefited from intermixed exposure, children with ADHD relied proportionally more on an implicit, nonspecific sense of “difference” than on an explicit representation of the critical feature.

An important consideration regarding these effects concerns statistical power. *Post hoc* power analysis using G*Power 3.1 (Faul et al., 2007) indicated adequate power for the main I/B effect: with *N* = 88 and *w* = 0.32, the power achieved was 1 − *β* = 0.85. However, the comparison involving explicit identification of the distinguishing feature had substantially lower power (1 − *β* = 0.52, w = 0.38, *N* = 28), reflecting the small sample size of the subset of correct responders in the INT conditions. To achieve adequate power of around 80% for an effect of this magnitude, approximately 54 correct responders in the INT conditions would be needed, which would imply a considerably larger overall study. Therefore, this aspect of the results should be considered a preliminary directional finding that would be worth replicating in the future.

Medication status showed no reliable association with either the likelihood of judging the stimuli as different on the Different-test-trial, *r*(42) = 0.050, *p* = 0.75, nor with the ability to specify the distinguishing feature once it had been detected, *r*(42) = −0.017, *p* = 0.91. These findings indicate that the perceptual-learning effects observed in the ADHD groups were independent of acute medication status.

#### 3.2.2 Same-test-trial

Finally, we analyzed performance on the Same-test-trial (right panel of Figure 2). As in adult same–different studies of the visual I/B effect (e.g., Lavis and Mitchell, 2006; Recio et al., 2016; Rodríguez and Rodríguez-San Juan, 2025), accuracy was near ceiling. We applied the same 2 × 2 × 2 log-linear model used for the Different-test-trial, with *ADHD condition* (ADHD vs. Control) × *exposure schedule* (INT vs. BLK) × *response accuracy* (Correct vs. Incorrect). The model of complete independence already fitted the data, G^2^(4) = 2.29, *p* = 0.68, and no lower-order main effects or interactions approached significance, largest G^2^ = 1.15, *p* = 0.28. Thus, Same-test-trial accuracy remained at ceiling irrespective of ADHD status or exposure schedule, extending the adult pattern to a much younger population—including children with ADHD—for the first time.

## 4 General discussion

This study provides the first evidence indicating that the perceptual learning mechanisms responsible for the intermixed-blocked effect function effectively in children and adolescents with ADHD. However, our results suggest the existence of important differences in the behavioral expression of these mechanisms compared to their typically developing peers. Our findings point to a dissociation between the implicit and explicit aspects of perceptual learning in ADHD, with important theoretical and practical implications.

Traditionally, it has been considered that improvement in the differentiation between stimuli after their intermixed pre-exposure is supported by automatic and involuntary mechanisms, described in terms of the modulation of the salience of the stimulus characteristics (and, at a representational level, of changes in the activation or inhibition of representational elements), which leads to greater ease in detecting distinctive features after alternating exposure to stimuli (e.g., Gibson, 1969; Hall, 2003; Hall and Rodríguez, 2019; McLaren and Mackintosh, 2000). In line with this interpretation, the first piece of our findings is that, in both groups (ADHD and control), intermixed pre-exposure facilitated the differentiation between two similar stimuli to a greater extent than blocked pre-exposure. This indicates that the fundamental mechanisms of perceptual learning function effectively in ADHD.

However, the expression of the intermixed–blocked effect differed between the control and ADHD conditions depending on the demands of the task. In the part of the test that involved only indicating whether the stimuli were the same or different, the performance of the ADHD group was comparable to that of the control group, and the advantage of intermixed pre-exposure was equivalent in both groups. In contrast, when the task also required explicitly identifying the difference between stimuli, the intermixed advantage in the ADHD group was reduced relative to that observed in the control group, which showed greater explicit awareness of the diagnostic distinguishing feature. This dissociation suggests that, at least in the intermixed-blocked paradigm, ADHD does not necessarily compromise the automatic processes of perceptual differentiation reflected in discrimination, but it does compromise conscious access to what has been learned and/or the ability to verbalize it.

These results strongly support models of executive function deficits in ADHD, while challenging the notion that implicit learning is broadly affected in this population. Previous inconsistent results in implicit learning research (e.g., Barnes et al., 2010; Domuta and Péntek, 2000; Karatekin et al., 2009; Rosas et al., 2010) may reflect methodological confounds rather than true learning deficits. Our perceptual learning paradigm, which minimizes executive demands during both learning and testing, reveals that automatic learning mechanisms, at least those involved in this type of perceptual task, continue to function in ADHD. The observed pattern suggests that children with ADHD can acquire perceptual knowledge through implicit mechanisms but have difficulty translating this knowledge into an explicit and verbally accessible form. Converging evidence that implicit learning on perceptual information may be preserved in ADHD comes from Gaschler et al. (2022), who showed that adults with ADHD incidentally learned covariation regularities between visual features and could use that implicit knowledge to guide their responses with accuracy comparable to that of controls.

Understanding that implicit learning appears to remain largely intact in ADHD has important practical consequences. A crucial insight from our findings is that children with ADHD may fail to report perceptual differences that they are in fact capable of detecting. This distinction should be considered by educational professionals when assessing student understanding and designing evaluation methods. Traditional assessment approaches that rely heavily on verbal articulation of differences or explicit feature identification may underestimate the perceptual competencies of students with ADHD.

Additionally, our results highlight the well-established benefits of exposure conditions that facilitate stimulus comparison. The intermixed presentation schedules that proved effective in our study can be exploited to enhance differentiation in diverse applied contexts. Educational materials and training protocols could incorporate alternating presentations of similar concepts, patterns, or stimuli to promote better discrimination learning, taking advantage of these preserved implicit mechanisms while reducing demands on explicit verbalization.

Our procedure offers a promising tool for assessing the dissociation between implicit and explicit learning in neurodevelopmental disorders. Unlike previous paradigms, which may confound learning with performance factors, perceptual learning tasks provide a clearer assessment of automatic learning mechanisms. This approach could be adapted to study other clinical populations in which executive-implicit dissociations are theoretically predicted.

Before concluding, it is necessary to point out several limitations of our study in order to identify avenues for future research. First, the sample we used was selected for convenience, which may limit the generalizability of the results, for example, with respect to other populations with ADHD with different severity profiles or comorbidities. Furthermore, although the study had adequate statistical power to detect the main intermixed-blocked effect, the analysis of the ability to explicitly identify the differentiating stimulus feature was based on a small subsample of participants. This resulted in modest statistical power for this comparison. Therefore, this finding should be considered preliminary until it is clearly replicated in the future. Also, as the sample was limited to children and adolescents, it remains to be determined whether the same dissociation between implicit and explicit perceptual learning extends to adults with ADHD. And the fact that a single paradigm and a single perceptual domain (visual stimuli) were used encourages examination of whether these results generalize to other types of stimuli and perceptual tasks. Finally, the study did not include measures of EF components, which would have allowed for a more precise characterization of which subprocesses (such as working memory capacity, inhibitory control, or metacognitive monitoring) mediate in the observed pattern of results. Investigating whether explicit training can improve conscious access to implicitly learned information would also provide useful information for intervention strategies. The inclusion of such measures in future studies would allow for a more robust testing of the EF deficit hypothesis.

Despite all these limitations, the current results contribute to a more nuanced understanding of cognitive abilities in ADHD and open up new avenues for educational and therapeutic interventions. Future research addressing the limitations noted above will help to determine the scope and robustness of the results observed here.

## Data Availability

The datasets presented in this study can be found in online repositories. The names of the repository/repositories and accession number(s) can be found at https://osf.io/76fqn/.
